# Amino acid variation at VP1-145 of enterovirus A71 determines the viral infectivity and receptor usage in a primary human intestinal model

**DOI:** 10.3389/fmicb.2023.1045587

**Published:** 2023-04-17

**Authors:** Ikrame Aknouch, Inés García-Rodríguez, Francesca Paola Giugliano, Carlemi Calitz, Gerrit Koen, Hetty van Eijk, Nina Johannessson, Sjoerd Rebers, Lieke Brouwer, Vanesa Muncan, Koert J. Stittelaar, Dasja Pajkrt, Katja C. Wolthers, Adithya Sridhar

**Affiliations:** ^1^Department of Medical Microbiology, OrganoVIR Labs, Amsterdam UMC, Location Academic Medical Center, University of Amsterdam, Amsterdam, Netherlands; ^2^Department of Pediatric Infectious Diseases, Emma Children’s Hospital, Amsterdam UMC, Location Academic Medical Center, University of Amsterdam, Amsterdam, Netherlands; ^3^Viroclinics Xplore, Schaijk, Netherlands; ^4^Tytgat Institute for Intestinal and Liver Research, Amsterdam Gastroenterology Endocrinology and Metabolism, Amsterdam UMC, Academic Medical Center, University of Amsterdam, Amsterdam, Netherlands; ^5^Department of Epidemiology, Bioinformatics and Animal Models, Wageningen Bioveterinary Research, Wageningen University, Wageningen, Netherlands

**Keywords:** EV-A71, VP1-145, heparin sulfate proteoglycan, human inestinal organoids, polarized epithelium, Transwell

## Abstract

Enterovirus A71 (EV-A71) can elicit a wide variety of human diseases such as hand, foot, and mouth disease and severe or fatal neurological complications. It is not clearly understood what determines the virulence and fitness of EV-A71. It has been observed that amino acid changes in the receptor binding protein, VP1, resulting in viral binding to heparan sulfate proteoglycans (HSPGs) may be important for the ability of EV-A71 to infect neuronal tissue. In this study, we identified that the presence of glutamine, as opposed to glutamic acid, at VP1-145 is key for viral infection in a 2D human fetal intestinal model, consistent with previous findings in an airway organoid model. Moreover, pre-treatment of EV-A71 particles with low molecular weight heparin to block HSPG-binding significantly reduced the infectivity of two clinical EV-A71 isolates and viral mutants carrying glutamine at VP1-145. Our data indicates that mutations in VP1 leading to HSPG-binding enhances viral replication in the human gut. These mutations resulting in increased production of viral particles at the primary replication site could lead to a higher risk of subsequent neuroinfection.

**Importance:**

With the near eradication of polio worldwide, polio-like illness (as is increasingly caused by EV-A71 infections) is of emerging concern. EV-A71 is indeed the most neurotropic enterovirus that poses a major threat globally to public health and specifically in infants and young children. Our findings will contribute to the understanding of the virulence and the pathogenicity of this virus. Further, our data also supports the identification of potential therapeutic targets against severe EV-A71 infection especially among infants and young children. Furthermore, our work highlights the key role of HSPG-binding mutations in the disease outcome of EV-A71. Additionally, EV-A71 is not able to infect the gut (the primary replication site in humans) in traditionally used animal models. Thus, our research highlights the need for human-based models to study human viral infections.

**Graphical Abstract:**
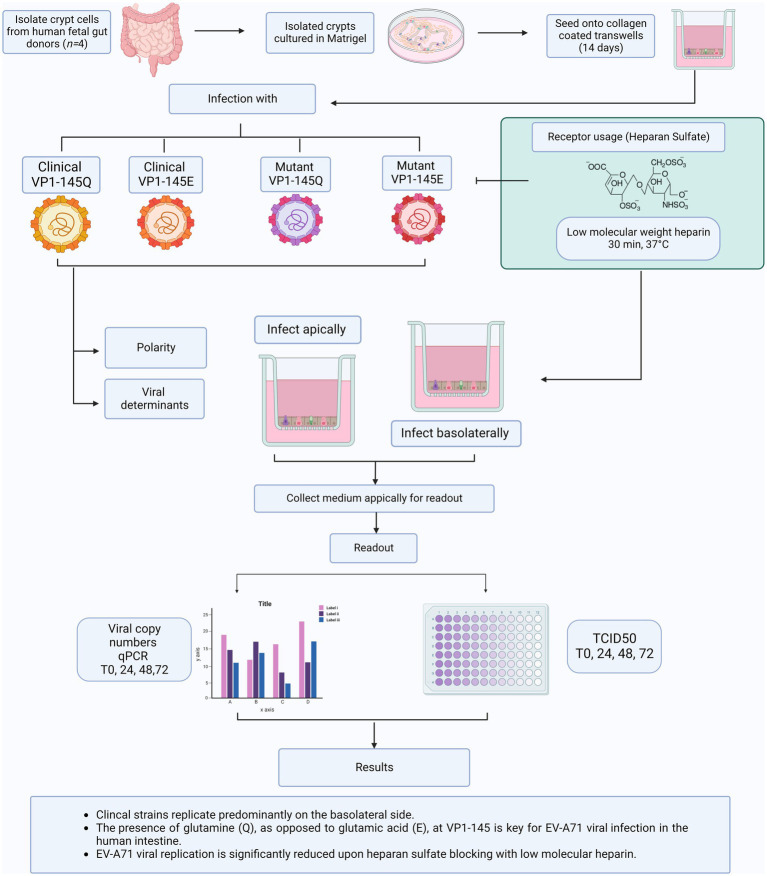

## 1. Introduction

Enterovirus A71 (EV-A71), belonging to the genus Enterovirus within the family *Picornaviridae*, is a major human pathogen that causes large outbreaks of hand, foot, and mouth disease (HFMD) in the Asia-Pacific region (Lin et al., 2002). In most cases, it is a self-limiting infection but EV-A71 is also associated with severe neurological disorders, such as meningitis, encephalitis, and polio-like paralysis resulting in high fatalities (up to 7.9%) among infants and young children (Ooi et al., 2010; Ryu et al., 2010; Solomon et al., 2010). With the near eradication of polio worldwide, polio-like illness is a concern, making EV-A71 the most prevalent neurotropic enterovirus posing a major threat globally to public health (Solomon et al., 2010; Xing and Morigen, 2014).

Viral determinants driving EV-A71 neurotropism are not well understood. One possibility for an expanded neuronal tissue tropism is through mutations in the receptor binding capsid protein, VP1, resulting in virus attachment or entry through additional receptors. In this regard, multiple cellular receptors have been identified for EV-A71, including human scavenger receptor class B2 (SCARB2) (Yamayoshi et al., 2009), P-selectin glycoprotein ligand-1 (PSGL-1) (Nishimura et al., 2009), sialylated glycan (Yang et al., 2009; Su et al., 2012), annexin II (Yang et al., 2011), vimentin (du et al., 2014), and nucleolin (Su et al., 2015). Furthermore, heparan sulfate proteoglycans (HSPGs) that are widely expressed on all cell types have been suggested as an attachment receptor for EV-A71. Illustratively, a single amino acid change in EV-A71 VP1 region results in HSPG-binding due to changes in surface charge of the virions (Tan et al., 2013). The positive charge resulting from glutamine (Q) on VP1-145, among other sites (lysine at 97, 98, 162, 242, and 244), constitutes a major HSPG-binding site in contrast to the negative charge resulting from glutamic acid (E) at the same residue (Byrnes and Griffin, 2000; Tan et al., 2017).

Clinical and epidemiological data indicate that these HSPG-binding mutations may be important for disease outcome of EV-A71. Indeed, isolates with 145Q are associated with severe disease in humans (Chang et al., 2012; Huang et al., 2012; Tseligka et al., 2018). Furthermore, Tseligka et al. (2018) isolated an EV-A71 strain with lysine at VP1-97 from stool, blood, and cerebrospinal fluid of an immunocompromised patient and demonstrated usage of HSPG by this strain in various cell lines and reconstituted respiratory, intestinal, and neural tissues. While their clinical and *in vitro* findings support the importance of HSPG-binding for EV-A71 virulence (Tseligka et al., 2018), data from animal studies in cynomolgus monkeys and mice are contradictory. In particular, the mutations most frequently associated with neurological disease, VP1-145Q/G, have reduced neurovirulence in both monkey and mice models compared to the non-HPSG binding mutation VP1-145E (Chua et al., 2008; Kataoka et al., 2015; Fujii et al., 2018).

To understand whether the differences between animal and clinical data are merely based on differences in observations or in mechanisms of disease, we previously used human airway organoid models to characterize virulence of VP1-145Q and VP1-145E variants. In human airway organoid models, VP1-145Q was more virulent than VP1-145E (van der Sanden et al., 2018). Furthermore, Tseligka et al. indicated that selection of HSPG-binding mutations in the intestine might play a key role in viral dissemination to the CNS. However, to date, there has not been a thorough characterization on the role of HSPG-binding mutations in the primary replication site, namely the intestine (Tseligka et al., 2018). Therefore, in this study, we further expand on this observation by assessing the virulence of both EV-A71 clinical isolates and mutants with either a Q or E at VP1-145 using physiologically relevant primary human fetal organoids model of the human intestine, which is the primary replication site of EV-A71. We previously demonstrated the intestine organoid models are susceptible to EV-A71 infection (Roodsant et al., 2020). These human intestinal organoids, which are extensively characterized and previously applied for other viral pathogens, closely recapitulate the complex multicellular composition and architecture of the human intestine providing an opportunity to study host-pathogen interactions in the human context (García-Rodríguez et al., 2020, 2021; Sridhar et al., 2020; Aknouch et al., 2022). Finally, we evaluate the dependence on HSPG of the different EV-A71 strains in this human intestinal organoid model.

## 2. Materials and methods

### 2.1. EV-A71 and EV-A71 mutants generation

Enterovirus A71 (EV-A71) clinical strains C1-91-480-Q (accession # AB524200) and C1-1185-E (accession # AB524169) were kindly provided by the National Institute for Public Health and the Environment, Bilthoven, the Netherlands. These strains were isolated from clinical specimens as part of the national enterovirus surveillance system in the Netherlands between 1991 and 2007. EV-A71 strains were propagated in RD99 cells maintained in Eagle’s minimum essential medium (EMEM; Lonza) supplemented with 8% heat-inactivated fetalbovine serum (HI-FBS; Sigma-Aldrich), 100 U-mg/mL penicillin- streptomycin (Lonzo Bio Whittaker), 1% non-essential amino acids (NEAA; ScienCell Research Laboratories) and 200 nM L-glutamine (Lonza). The 50% tissue culture infectious dose (TCID50) of virus stocks was calculated according to the method of Reed and Muench (Reed and Muench, 1938).

The preparation of VP1-145 mutants was performed according to the previous study (van der Sanden et al., 2018). Previously constructed full-length genomic cDNA clones of the clinical isolate 02363-Thai-02 (accession # AB747375.1) was used to introduce amino acid mutations at VP1-145 by site-directed mutagenesis using PCR (Nishimura et al., 2009). Linearized clones were transcribed with T7 RNA polymerase (MEGAscript T7 Kit, Ambion) following manufacturer’s instructions. The RNA was transfected into RD cells using a Lipofectamine 2000 reagent (Life Technologies) following the protocol of the manufacturer. Cells were incubated at 37°C,5% CO_2_ until a clear cytopathic effect (CPE) was visible. Cells were freeze–thawed three times to release viral particles, and viruses were amplified once more in fresh RD99 cells. The infectious viral particle amounts in the supernatants were determined as described above, and mutation introduction was verified by sequencing complete capsid-encoding regions. To characterize mutant viral growth, RD99 cells were grown to 90% confluency in 96-well plates and infected with the mutant viruses at a multiplicity of infection of 0.3 for 30 min at 37°C, 5% CO_2_. Infected cells were subsequently washed twice with PBS and incubated at 37°C, 5% CO_2_ in 100 μL of EMEM supplemented with 10% fetal calf serum. Viral titers were determined by virus titration assay using RD99 cells, as previously described (van der Sanden et al., 2018). Viral titers were expressed as TCID50.

### 2.2. Human fetal intestinal organoids

#### 2.2.1. Ethics statement

Human fetal intestinal tissue, gestational age 19–20 weeks, was obtained by the HIS Mouse Facility of the Amsterdam University Medical Center (AUMC), with a written informed consent obtained from all donors for the use of the material for research purposes. The human fetal intestinal tissue was obtained with approval of the medical ethical committee of the AUMC, together with approval of the experimental procedures by the HIS Mouse Facility. All experiments were performed according to the relevant guidelines and regulations, mentioned in the Amsterdam UMC Research Code regulations and in accordance with Dutch law.

#### 2.2.2. Culture of human fetal intestinal organoids and monolayers culture

For the generation of fetal intestinal organoids, crypts were isolated from fetal small intestinal tissue as described previously (Sato et al., 2009; Roodsant et al., 2020). Isolated crypts were resuspended in Matrigel® (Corning) and dispensed in three 10 μL droplets per well in a 24-well tissue culture plate. The Matrigel® domes were allowed to crosslink at 37°C with 5% CO_2_ followed by the addition of 500 μL Human IntestiCult™ Organoid Growth Medium (HIOGM, Stemcell™ Technologies) supplemented with 100 U-mg/mL penicillin–streptomycin (Gibco, Thermo Fischer Scientific). Organoid cultures were incubated at 37°C, 5% CO_2_. Medium was changed every 2–3 days and organoids were passaged every 6–10 days as described previously (Roodsant et al., 2020) or by enzymatic dissociation as described below for seeding onto cell culture inserts.

Fetal organoid-derived monolayers were cultured on Transwell® cell culture inserts (6.5 mm, 3.0 μm pore size, VWR) as described previously (Roodsant et al., 2020). Briefly, Transwell® inserts were coated with 100 μL of 20 μg/mL collagen type I (rat tail, Ibidi) in 0.01% (v/v) acetic acid for 1 hour at room temperature (RT) and washed twice with PBS before use. Human fetal organoids were collected, and single cell suspension was obtained by treatment with TryplE™ (Gibco, Thermo Fischer Scientific) for 10 min at 37°C with 5% CO_2_. Cells were diluted to 10^6^ cells/mL and 100 μL of cell suspension was seeded per insert. For the first 3 days, the monolayers were cultured in IntestiCult™ Organoid Growth Medium (HIOGM, Stemcell™ Technologies) with 10 μM Y-27632 (Sigma-Aldrich, St. Louis, United States), after which medium was changed to IntestiCult™ Organoid Growth Medium (HIOGM, Stemcell™ Technologies) from day 7 organoid growth medium was replaced with differentiation medium, which is a 1:1 mixture of the Basal Component of IntestiCult™ (Stemcell™ Technologies) and Advanced Dulbecco’s Modified Eagle Medium/Nutrient Mixture F-12 (DMEM/F12, Gibco, Thermo Fisher Scientific, Waltham, USA) supplemented with 100 U/mL Pen-Strep, 7.5 mM HEPES (Sigma-Aldrich, St. Louis, United States) and 0.5× Glutamax (Thermo Fisher Scientific, Waltham, USA). Medium was refreshed every 2–3 days. To monitor monolayer formation, trans-epithelial electrical resistance (TEER) was measured at days 3, 7, 10, and 14 after seeding with a EVOM-2 voltohmmeter (World Precision Instruments, Sarasota, USA). Resistance values were calculated using an empty Transwell® and multiplied by the surface area of the insert to obtain the TEER (Ω × cm2).

### 2.3. Infection of fetal intestinal organoid-derived monolayers with EV-A71 strains

Fetal intestinal organoid monolayers were infected apically or basolaterally with 10^5^ TCID50/100 μL (basolateral infection were initiated by adding the virus to the media). After 2 h incubation at 37°C with 5% CO_2_, unbound virus was removed by washing three times with PBS and fresh differentiation medium was added. The monolayers were incubated for 10 min, after which the 0-h time point was collected, removing 100 μL from the apical and the basolateral side. At 24-, 48-, and 72-h post infection, a 100 μL sample was taken from the apical and the basolateral compartment and the collected volume was replaced with fresh differentiation medium.

### 2.4. Blocking of HSPG-binding of EV-A71 in fetal intestinal organoid-derived monolayers

To evaluate the involvement of HSPG-dependent replication by different EV-A71 strains, HSPG-binding sites on the viruses were blocked with low molecular weight heparin (LMWH; H4784, Sigma Aldrich). EV-A71 strains, 10^5^ TCID50/100 μL, were pre-incubated with 5 mg/mL LMWH for 30 min at 37°C with 5% CO_2_. Subsequently 100 μL or 600 μL of the virus/LMWH mixtures were, respectively, added to the apical or basolateral compartment of the Transwell® inserts. The monolayers were incubated for 2 h at 37°C with 5% CO_2_ and subsequently washed three times with PBS. Fresh differentiation medium was added, and the monolayers were incubated for 10 min, after which the 0-h time point was collected, removing 100 μL from the apical and the basolateral side. At 24-, 48-, and 72-h post infection, a 100 μL sample was taken from the apical and the basolateral compartment and the collected volume was replaced with fresh differentiation medium.

### 2.5. EV-A71 quantification by viral RNA detection and virus titration

To evaluate the replication kinetics of different EV-A71 strains, the collected supernatants were tested for the presence of EV-A71 RNA using reverse transcription quantitative polymerase chain reaction (RT-qPCR) while virus titration by Reed- Muench method was used to evaluate the presence of EV-A71 infectious virus particles (Reed and Muench, 1938).

#### 2.5.1. EV-A71 viral RNA detection

Briefly, RNA extraction was performed from apical and basolateral medium samples using the Bioline Isolate II RNA mini kit (Meidian Bioscience®, Cincinatti, United States) according to manufacturer’s protocol. Viral RNA was eluted in 60 μL elution buffer and reverse transcribed using the SupersCript II kit according to manufacturer’s instructions. Five microliters of cDNA were used for RT-qPCR and performed on CFX Connect Real-Time PCR Detection System (Bio-Rad, California, USA) using the SYBR Green I Master kit (Roche Diagnostics). The quantitative cycle (Cq) values were transformed into viral copies using EV-A71 standard curve with known concentrations of the viral genome. Primer sequences are listed in Supplementary Table 1.

#### 2.5.2. EVA-71 virus titration

The presence of EV-A71 infectious viral particles in the collected medium samples was detected by virus titration on RD99 cells. Briefly, EV-A71 was cultured on RD99 cells maintained in Eagle’s minimum essential medium (EMEM; Lonza) supplemented with 8% HI-FBS(Sigma-Aldrich), 100 U-mg/mL penicillin- streptomycin (Lonzo Bio Whittaker), non-essential amino acids (NEAA; ScienCell Research Laboratories) and 200 nM L-glutamine (Lonza). The TCID50 of virus stocks and samples was calculated according to the method of Reed and Muench (Reed and Muench, 1938).

#### 2.5.3. Sequencing of EV-A71 strains

Nucleic acids were extracted from all samples as reported previously (Boom et al., 1990). The complete VP1 region was sequenced for typing (Nix et al., 2006). Briefly, a nested PCR was conducted using primers VP1 222, 224, AN88, and AN89. The PCR products were analyzed by agarose gel electrophoresis. Positive samples with a PCR fragment size of 400 base pairs (bp) were included for sequencing. The sequencing reaction was performed using a Big Dye Terminator Kit. Sample sequences were assembled in CodonCode Aligner (version 6.0.2) and aligned with Aliview software^1^ using the MUSCLE method. Maximum-likelihood (ML) phylogenetic trees including all sample strains and reference strain from the GenBank database were constructed for the VP1 sequence (nt positions 2,581 to 2,934) of the reference genome sequence of the Thai-02 EV-A71 strain [AB747375.1, accession no. 02363]. Primers sequences are listed in Supplementary Table 2.

#### 2.5.4. Statistical analysis

Graphs and statistics were performed using GraphPad prism 8 (GraphPad Software, Inc., San Diego, California United States, www.graphpad.com). Organoids derived from four different fetal donors were used for all experiments resulting in four biological replicates (n = 4). All experiments were performed in duplicates (technical replicates) for each donor and geometric mean ± standard error of the mean (SEM) of biological replicates was plotted, unless stated otherwise, as indicated in figure legends. The fold increase in RNA copies was calculated by dividing the RNA copies of a specific time point to the average RNA copies at the 0-h time point. The effect of infection was tested with a ratio-paired t-test (one side), with value of *p* <0.05 considered statistically significant.

## 3. Results

### 3.1. VP1-145Q determines increased infectivity in the human fetal intestinal organoid-derived monolayers

To assess the replication kinetics of EV-A71 in the human intestine, human fetal organoid-derived monolayers were infected with EV-A71 clinical isolates C1-91–480 and C1-1185 with either a Q or E at VP1-145, respectively. The monolayers were infected either apically or basolaterally with a multiplicity of infection (MOI) of 0.01. Virus shedding was demonstrated in the apical compartment and was absent or low in the basolateral compartment (data not shown), as measured at several time points post infection and quantified by RT-qPCR and TCID50.

**Figure 1:**
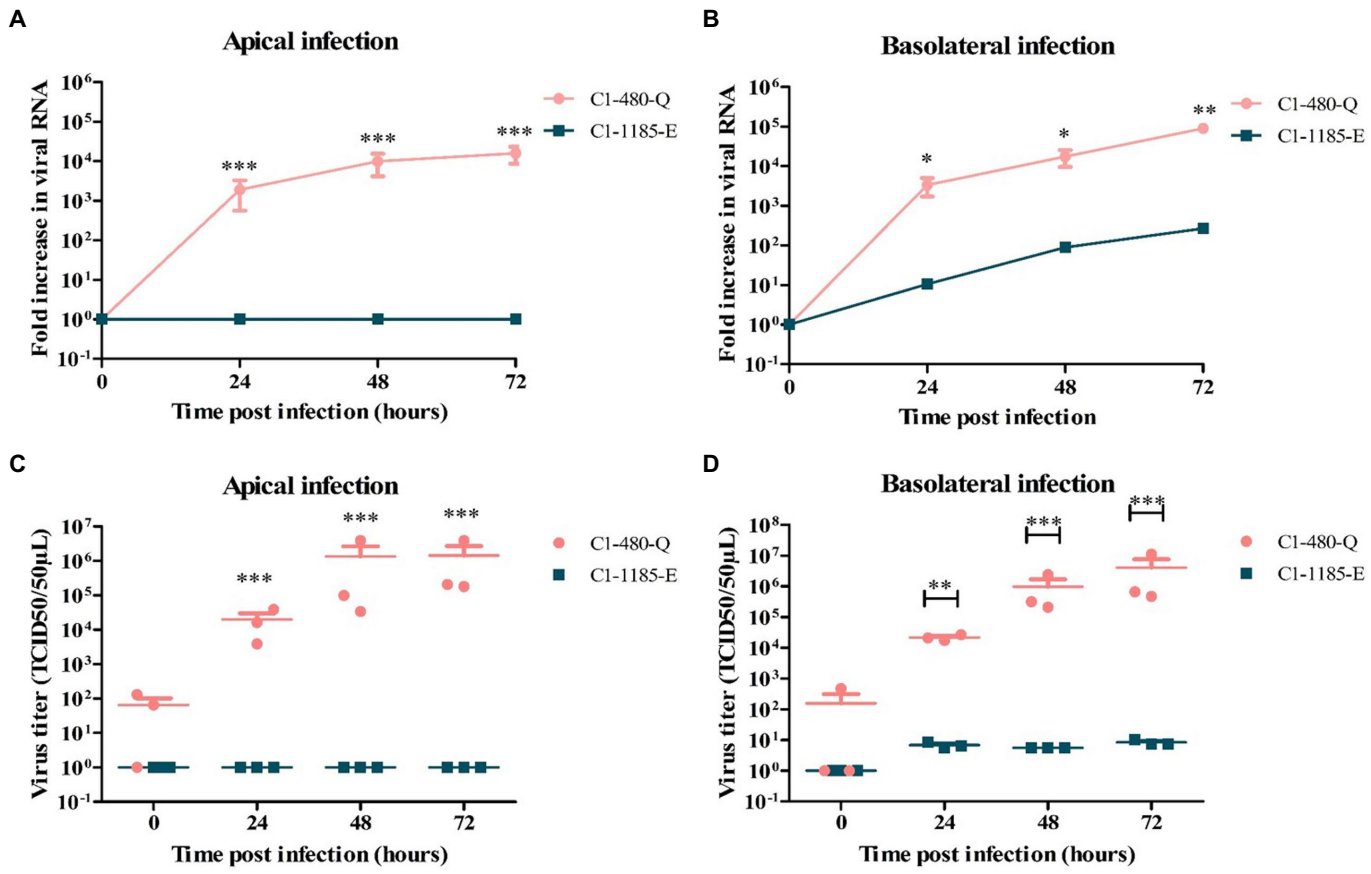
Replication kinetics of EV-A71 clinical isolates C1-480-Q and C1-1185-E in fetal intestinal organoid-derived monolayers. Fold increase in viral RNA copies and virus titers in media collected at different time points from the apical surface after apical **(A,C)** and basolateral **(B,D)** inoculation, respectively. In all cases, data represent the geometric mean ± SEM of four biological replicates with two technical replications per biological replicate. * value of *p* < 0.05; ** value of *p* < 0.01; *** value of *p* < 0.001.

Analysis of viral RNA showed that C1-480-Q clinical isolate replicated more efficiently than the C1-1185-E clinical isolate after apical (Figure 1A) and basolateral inoculation (Figure 1B). Additionally, C1-480-Q clinical isolate was able to infect the monolayers from both apical and basolateral compartments while C1-1185-E was infectious from the basolateral compartment only. The release of both clinical isolates primarily occurred apically.

To verify the generation of infectious viral particles, TCID50 analysis in RD99 cells was performed and the titers at several time points were determined. In line with the qPCR data, C1-480-Q clinical isolate showed a significant increase in viral titer irrespective of the route of inoculation, however, C1-1185-E replicated only after basolateral inoculation (Figures 1C,D).

### 3.2. VP1-145Q is a determinant of increased infectivity in viral mutants generated by site-directed mutagenesis

**Figure 2:**
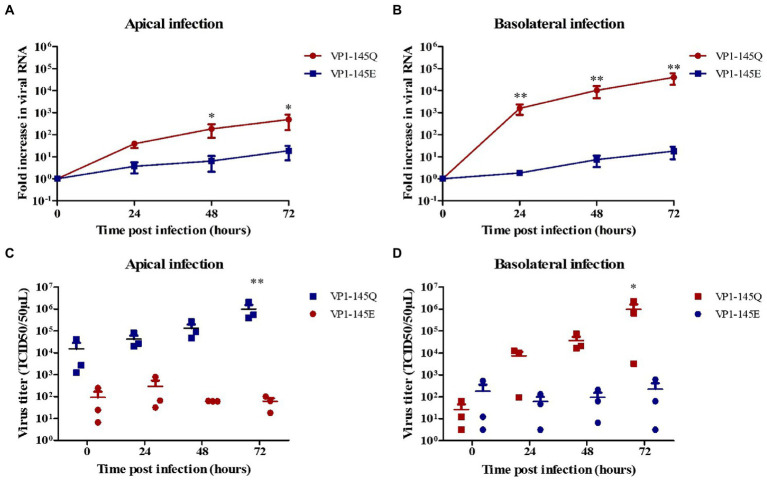
Replication kinetics of EV-A71 mutants VP1-145Q and VP1-145E in fetal intestinal organoid-derived monolayers. Fold increase in viral RNA copies and virus titers in the media collected at different time points from the apical surface after apical **(A,C)** and basolateral **(B,D)** inoculation, respectively. In all cases, data represent the geometric mean ± SEM of four biological replicates with two technical replications per biological replicate. * value of *p* < 0.05; ** value of *p* <0.01.

Further, to confirm VP1-145 as an infectious determinant in fetal intestinal organoid-derived monolayers, site-directed mutagenesis was performed to generate two EV-A71 mutants, with E (VP1-145E) or Q (VP1-145Q) amino acid at VP1-145. Like the previous observations using the clinical strains, the EV-A71 VP1-145Q mutant replicated more efficiently after both apical or basolateral inoculation compared to the VP1-145E mutant, which replicated, albeit poorly, after apical and basolateral inoculation (Figures 2A,B). Furthermore VP1-145Q mutant entry preferentially occurred *basolateral* with the release of the virus predominantly occurring apically. Additionally, the release of infectious viral particles was only measured for the VP1-145Q mutant and was found to be consistent with the RT-qPCR data (Figures 2C,D).

**Figure 3:**
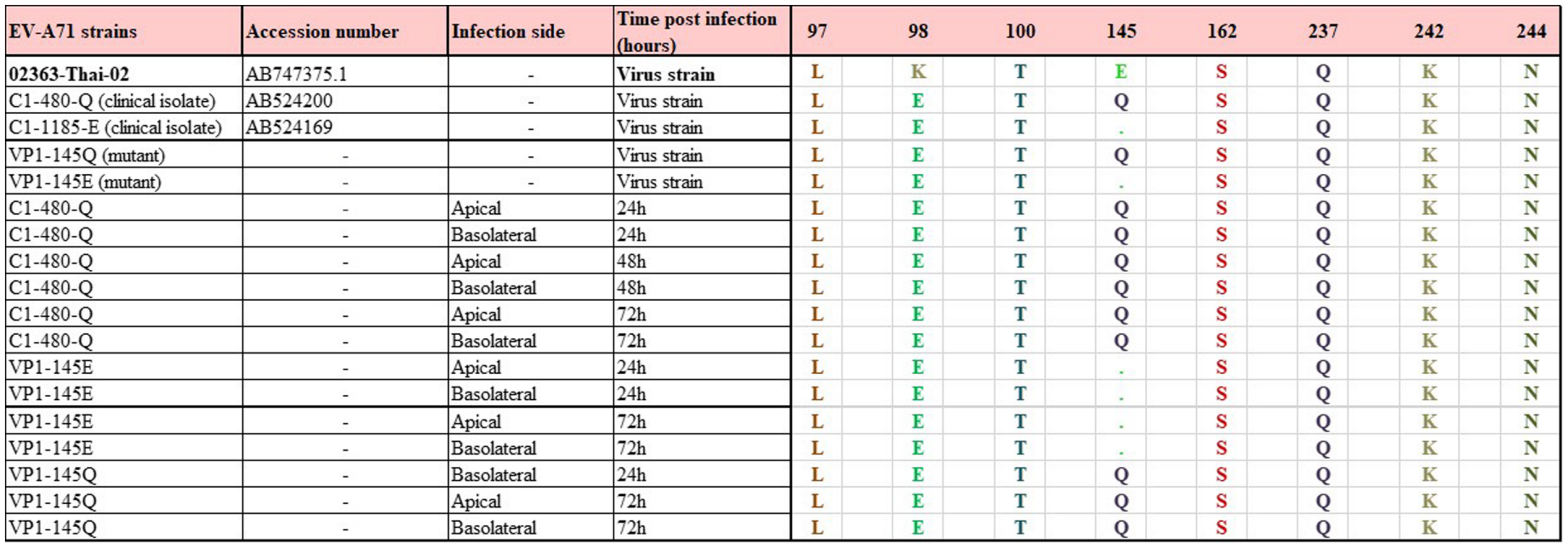
Representation among VP1 amino acid sequences of the EV-A71 mutant and clinical isolate strains and collected media from the infected monolayers. VP1 amino acid sequences were aligned to Enterovirus A71 genomic RNA complete genome strain: 02363-Thai-02 (accession number AB747375.1). Only qPCR positive samples were included. No changes and no adaptations were observed before and over the course of infection.*Sample ID used in the Supplementary Figure S3.

The complete capsid-encoding regions of EV-A71 clinical isolates, viral mutants, and different samples (before and after infection), were sequenced to verify the presence of the correct VP1-145 residue and to exclude residue mutations elsewhere in VP1 region. VP1 sequences were successfully obtained from all included samples and VP1-145 remained unchanged during infection (Figure 3).

### 3.3. VP1-145 is responsible for the differential sensitivity of EV-A71 isolates to heparin inhibition

Next, we evaluated the dependence on HSPG of the different EV-A71 strains in the human intestinal organoid-derived monolayers. To determine this, C1-91-480-Q and C1-1185-E clinical isolates were pre-treated with low molecular weight heparin (LMWH). The virus-LMWH mixture was added to the monolayers either apically or basolateral.

**Figure 4:**
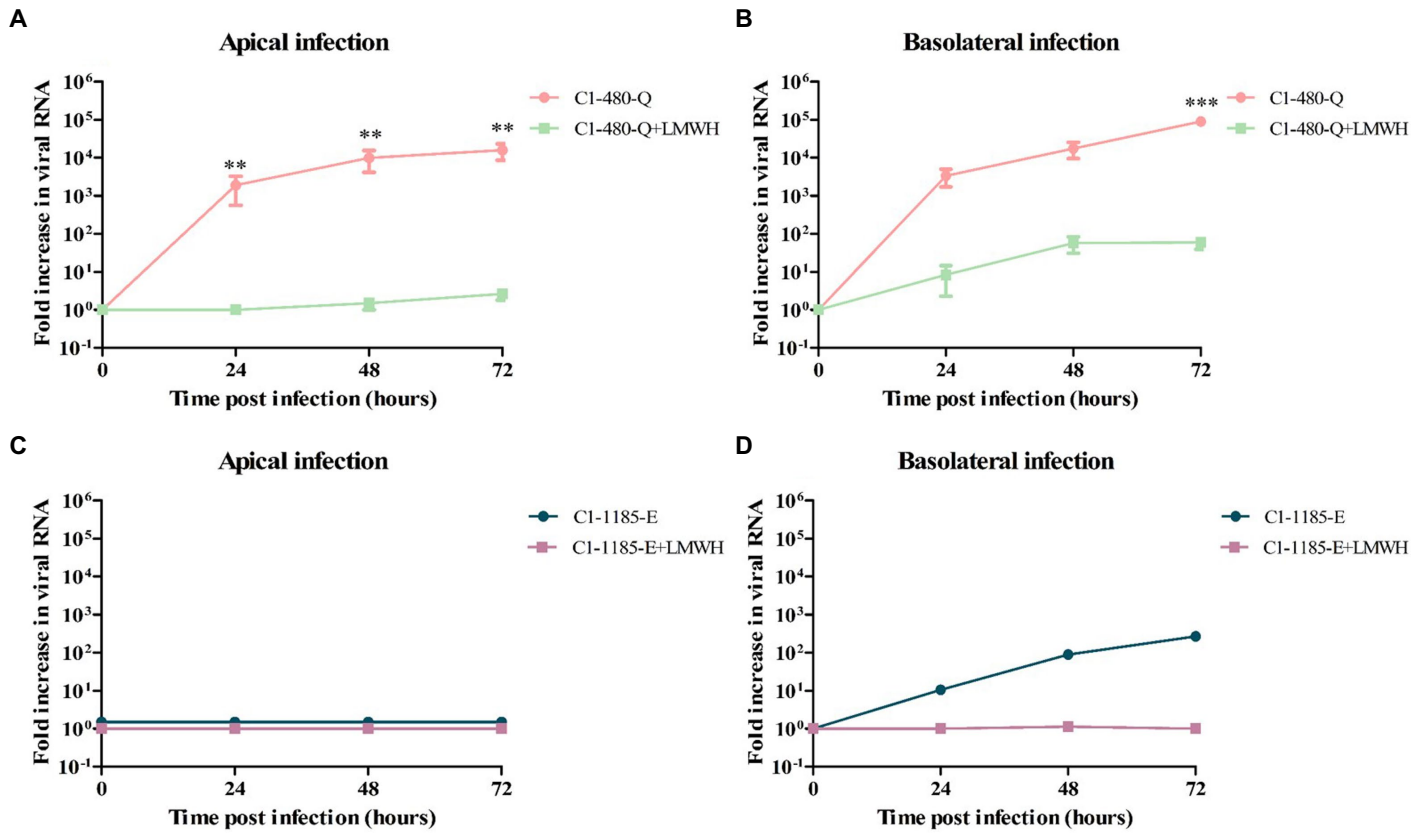
The susceptibility of C1-480-Q and C1-1185-E to HSPG-binding in fetal intestinal organoid-derived monolayers. **(A–D)** Fold increase in viral RNA in media collected at different time points from the apical compartment after apical **(A,C)** and basolateral **(B,D)** inoculation with and without pre-treatment with LMWH. In all cases, data represent the geometric mean ± SEM of four biological replicates with two technical replications per biological replicate. ** value of *p* <0.01; *** value of *p* <0.001.

As shown in Figure 4, the level of C1-91-480-Q infection was significantly reduced after LMWH pre-treatment at both apical and basolateral surface. In contrast, C1-1185-E virus replication was reduced after LMWH pre-treatment, however this reduction was not significant (Figure 4).

To verify the generation of infectious viral particles, TCID50 on RD99 cell lines was performed and the titers at several time points were determined. In line with the qPCR data, the C1-480-Q clinical isolate showed a significant reduction in viral titer after LMWH pre-treatment, irrespective of the route of inoculation (Supplementary Figure 1).

**Figure 5:**
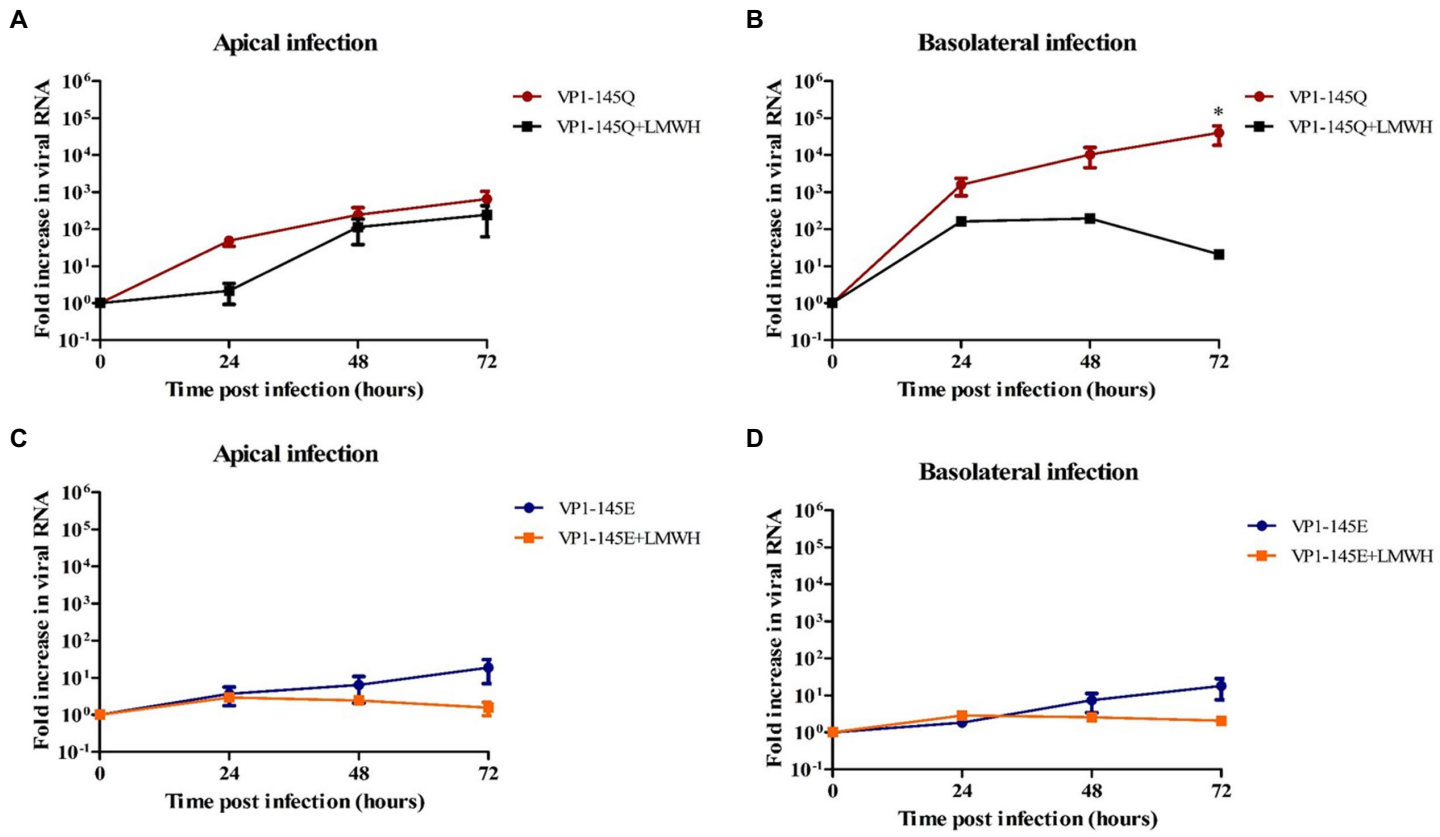
The susceptibility of EV-A71 mutants VP1-145Q and VP1-145E to HSPG-binding in fetal intestinal organoid-derived monolayers. **(A–D)** Fold increase in viral RNA in media collected on different time points from the apical surface after apical **(A,C)** and basolateral **(B,D)** inoculation with and without pre-treatment with LMWH. In all cases, data represent the geometric mean ± SEM of four biological replicates with two technical replications per biological replicate. *value of *p* <0.05.

Further to verify the role of VP1-145 in heparin binding, we then pre-treated EV-A71 VP1-145Q and VP1-145E mutants with LMWH. Similarly, to what was observed for EV-A71 clinical isolates, we found that LMWH significantly inhibited the level of VP1-145Q infection (Figures 5A,B). However, this significant reduction was not seen for the VP1-145E strain replication after LMWH pre-treatment (Figures 5C,D). This data was consistent with the viral titers (Supplementary Figure 2).

These results indicate that VP1-145 is a molecular determinant of EV-A71 infection in this fetal intestinal model and is linked to heparin binding.

## 4. Discussion

In this study, we demonstrated replication of EV-A71 clinical isolates and infectious mutants in human fetal intestinal organoid-derived monolayers. We identified that the presence of glutamine, as opposed to glutamic acid, at VP1-145 is key for EV-A71 infectivity in this model. Pre-treatment of EV-A71 viral particles with LMWH to block HSPG-binding significantly reduced infectivity of clinical isolates and viral mutants carrying glutamine at VP1-145.

Our data accentuate previous data showing VP1-145Q is a key determinant of EV-A71 infectivity in a human airway organoid model (van der Sanden et al., 2018). Here we show that VP1-145Q represented only ~9% of circulating human strains (Fujii et al., 2018) is a viral determinant of infectivity in the fetal intestinal organoid-derived monolayers. This is in line with previous studies showing that VP1-145Q variants are associated with increased disease severity in humans and are more frequently isolated in patients with severe neurological complications (Li et al., 2011; Zhang et al., 2014). In contrast, EV-A71 strains containing VP1-145E, which are highly dominant in patients (81%), replicated at a much lower level in both human intestinal organoid-derived monolayers and airway organoids. Thus, despite the lower replication efficacy in airway and intestinal models, EV-A71 strains containing VP-1-145E remain dominant. This could be due to the fact that non HSPG-binding EV-A71 strains with VP1-145E have been described to be more resistant to neutralizing antibodies than HSPG-binding viruses with VP1-145G (Tseligka et al., 2018). The emergence of more virulent HSPG-dependent mutants and their subsequent dissemination to the CNS could thus be favored in immunocompromised individuals with decreased antibody responses.

EV-A71 infection in our intestinal model was *via* the basolateral surface for most of the strains used in this study. Efficient infection of EV-A71 *via* the basolateral surface is in line with previous observations that the HSPG-binding strains of EV-A71 replicated more efficiently from the basolateral surface compared to non-HSPG strains (Tseligka et al., 2018). In contrast, Good *et al* demonstrated that the entry and exit of EV-A71 occurs apically in an enteroid model (Good et al., 2019). These contrasting observations could be due to differences between EV-A71 strains in the two studies. Different EV-A71 strains may have different binding efficiency, and this may modulate the infection outcome in various experimental set-ups. This is also supported in this study, showing that one of the strains, C1-480-Q, replicated efficiently at both sides. Additionally, this study was performed in four different donors, providing additional accuracy and reliability while the study by Good *et al* used only a single donor. Alternatively, organoid-derived monolayers could potentially differ in distribution of entry receptors, and polarization due to differences in protocols which could result in different replication kinetics and outcome of EV-A71 infection. In addition, varying distribution of HSPG can potentially influence EV-A71 entry and have an impact on the outcome of infection. In general, HSPGs are predominantly expressed at the basolateral surface of the human intestine cells, and this could explain higher replication of HSPG-binding strains at the basolateral surface (Oshiro et al., 2001).

In conclusion, our results demonstrate that EV-A71 can infect human fetal organoid-derived monolayers. We showed that the presence of Q, as opposed to E, at VP1-145 is key for viral infection in a fetal intestinal organoid-derived monolayer. Blocking virus access to cell surface HSPG leads to significant reduction of basolateral infection, suggesting that EV-A71 utilized cell surface heparan sulfate as an attachment receptor in this model. This indicates that HSPG constitutes an important mediator for EV-A71 infection, however, the exact mechanism of interaction of EV-A71 particles with negatively charged heparan sulfate on the cell surface remains unclear. Furthermore, our data indicates that HSPG-binding plays a key role in intestinal infection and selection of these HSPG-binding variants in the intestine might play an important role in viral dissemination to the CNS. Therefore, the functional relevance of heparin binding to viral pathogenesis in the CNS and the cooperative role of heparin binding with other known receptors should be further explored.

The determination of the molecular basis of the virus-receptor interaction in target cells will be useful for understanding the pathogenicity of EV-A71 and potential therapeutic targets against severe EV-A71 infection. Several strategies for prophylaxis that target HSPG are already being evaluated in other viruses including against human papillomavirus, herpes simplex virus, and influenza A virus (CARLUCCI et al., 2004; Lindahl, 2007; Cagno et al., 2015), and a similar strategy may be of value for EV-A71 infection.

## Data availability statement

The datasets presented in this study can be found in online repositories. The names of the repository/repositories and accession number(s) can be found below: NCBI GenBank [https://www.ncbi.nlm.nih.gov/genbank/], OP672343-OP672363.

## Ethics statement

Tissues were obtained with approval of the Ethical Committee of the Amsterdam UMC, together with approval of the experimental procedures by the HIS Mouse Facility (Amsterdam UMC). All methods were performed in accordance with the relevant guidelines and regulations, as stated in the Amsterdam UMC Research Code, in a certified laboratory (ISO15189 accreditation M304). The patients/participants provided their written informed consent to participate in this study.

## Author contributions

IA, AS, KS, DP, and KW: conceptualization and methodology. IA, AS, IG-R, HE, GK, NJ, FG, SR, and LB: investigation. AS, VM, KS, KW, and DP: funding. VM, AS, KS, KW, and DP: supervision and resources. KW and DP: project administration. IA: visualization. IA, AS, CC, KS, KW, and DP: writing – original draft. IG-R, FG, LB, HE, GK, NJ, and SR: writing – review and editing. All authors have read and agreed to the published version of the manuscript.

## Funding

This study was funded by Viroclinics Xplore, and OrganoVIR (grant 812673), and GUTVIBRATIONS (grant 953201).

## Conflict of interest

IA is employee of Viroclinics Xplore.

The remaining authors declare that the research was conducted in the absence of any commercial or financial relationships that could be construed as a potential conflict of interest.

## Publisher’s note

All claims expressed in this article are solely those of the authors and do not necessarily represent those of their affiliated organizations, or those of the publisher, the editors and the reviewers. Any product that may be evaluated in this article, or claim that may be made by its manufacturer, is not guaranteed or endorsed by the publisher.

## Footnotes

^1^ https://ormbunkar.se/aliview/

## Abbreviations

EV-A71, Enterovirus A71
HFMD, Hand, Foot, and Mouth Disease
SCARB2, Scavenger Receptor Class B2
PSGL-1, P-Selectin Glycoprotein Ligand-1
HSPGs, Heparan Sulfate Proteoglycans
LMWH, low molecular weight heparin
Q, glutamine
E, glutamic acid
K, lysine
R, arginine
2D, 2-dimensional
3D, 3-dimensional
BSA, Bovine serum albumin
CNS, Central nervous system
CPE, Cytopathic effect
DMEM/F12, Dulbecco’s Modified Eagle Medium/Nutrient Mixture F-12
dpi, Days post infection
EDTA, Ethylenediaminetetraacetic acid
EMEM, Eagle’s minimum essential medium
HI-FBS, heat-inactivated fetal bovine serum
HAE, Human airway epithelium
HuNoV, Human norovirus
LYZ, Lysozyme
M, Microfold
MOI, Multiplicity of infection
PBS, Phosphate Saline Buffer
qPCR, Quantitative Polymerase Chain Reaction
Cq, quantitative cycle
bp, base pairs
ML, maximum-likelihood
RT, Room temperature
Cytopathic effect, CPE
TCID50, 50% Tissue Culture Infectious Dose
TEER, trans-epithelial electrical resistance
NEAA, non-essential amino acids
SEM, standard error of the mean.
