# Supplementary material for: Amino acid variation at VP1-145 of enterovirus A71 determines the viral infectivity and receptor usage in a primary human intestinal model
Source: Front Microbiol. 2023 Apr 17;14:1045587. doi: 10.3389/fmicb.2023.1045587 (PMC10149690; doi:10.3389/fmicb.2023.1045587)
Supplement: Supplementary file 1 [file Table_1.DOCX]

**Supplementary data**

**Supplementary Table S1: List of primers used in RT-qPCR**

| **Virus** | **Forward primer (5’-3’)** | **Reverse primer (5’-3’)** | **NM number** |
| --- | --- | --- | --- |
| EV-A71 | GGCCCTGAATGCGGCTAAT | GGGATTGTCACCATAAGCAGCC | HA 11350962; HA1135096 |

**Supplementary Table S2: List of primers used in Sequencing**

| **Gene** | **Primers** | **Position** | **Sequence** |
| --- | --- | --- | --- |
| VP1 | 222 | 2969 | CICCIGGIGGIAYRWACAT |
| VP1 | 224 | 1977 | GCIATGYTIGGIACICAYRT |
| VP1 | AN88 | 2977 | TACTGGACCACCTGGNGGNAYRWACAT |
| VP1 | AN89 | 2602 | CCAGCACTGACAGCAGYNGARAYNGG |
