# Supplementary figures and images for: Amino acid variation at VP1-145 of enterovirus A71 determines the viral infectivity and receptor usage in a primary human intestinal model
Source: Front Microbiol. 2023 Apr 17;14:1045587. doi: 10.3389/fmicb.2023.1045587 (PMC10149690; doi:10.3389/fmicb.2023.1045587)

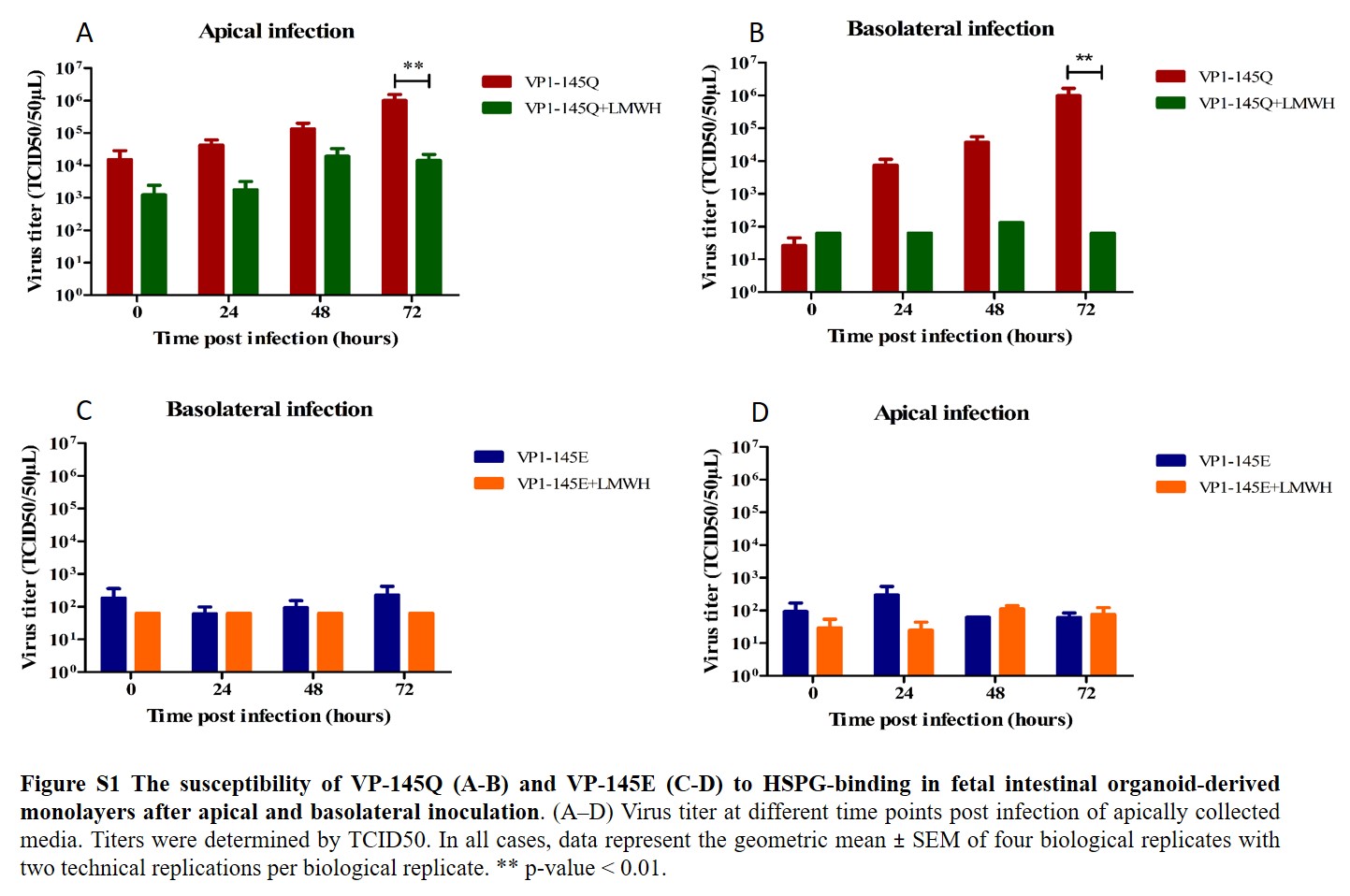

Supplement: Supplementary file 3 [file Image_1.jpg]

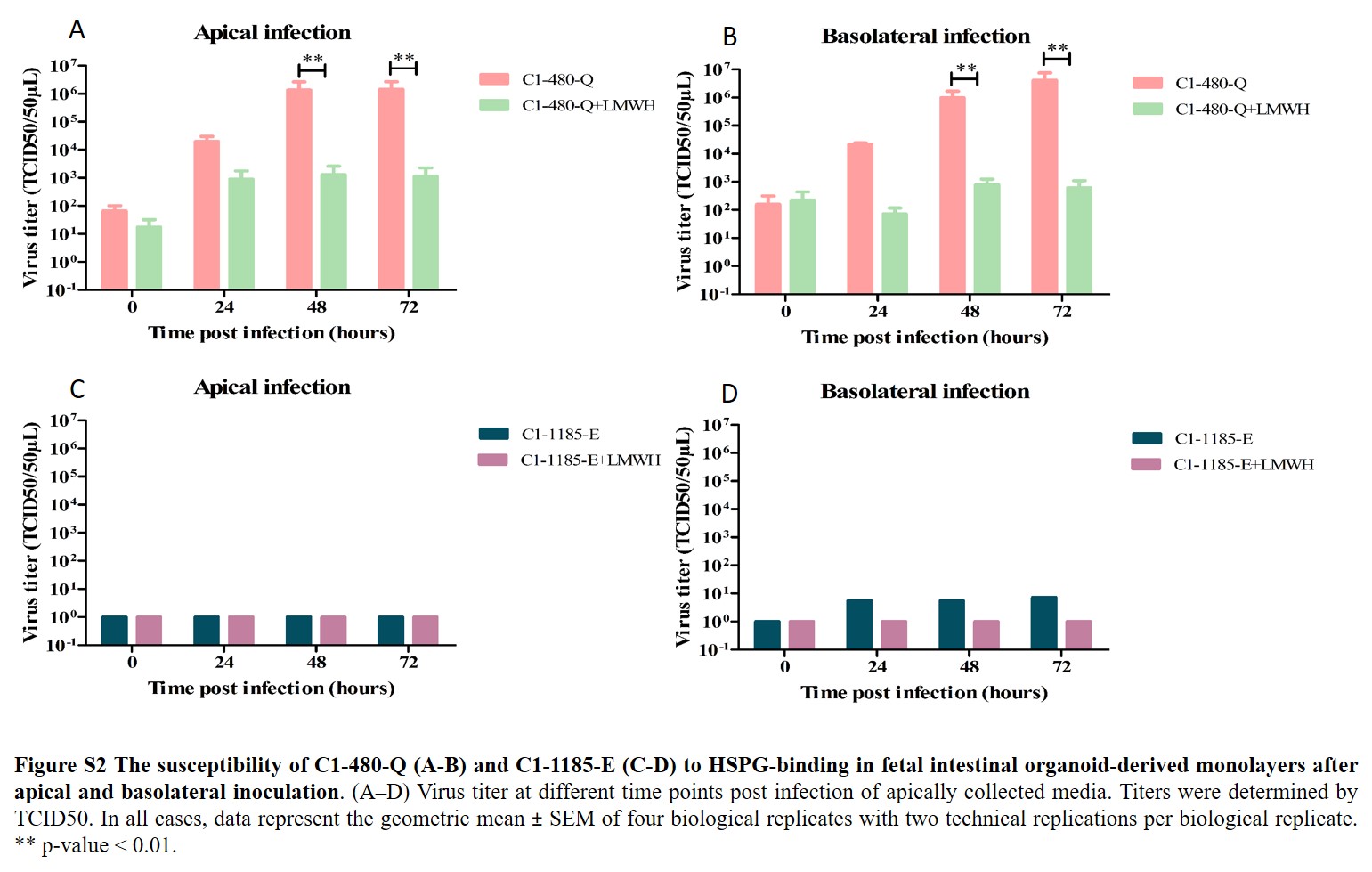

Supplement: Supplementary file 4 [file Image_2.jpg]
